# Greek Traditional Dances: A Way to Support Intellectual, Psychological, and Motor Functions in Senior Citizens at Risk of Neurodegeneration

**DOI:** 10.3389/fnagi.2019.00006

**Published:** 2019-01-25

**Authors:** Styliani Douka, Vasiliki I. Zilidou, Olympia Lilou, Magda Tsolaki

**Affiliations:** ^1^Laboratory of Sports, Tourism and Recreation Management, School of Physical Education and Sport Science, Aristotle University of Thessaloniki, Thessaloniki, Greece; ^2^Laboratory of Medical Physics, Medical School, Aristotle University of Thessaloniki, Thessaloniki, Greece; ^3^Department of Neurology, Medical School, Aristotle University of Thessaloniki, Thessaloniki, Greece

**Keywords:** Greek traditional dances, dementia, quality of life, physical health, mental health

## Abstract

One of the major problems that elderly people are facing is dementia. For scientist’s dementia is a medical, social and economic problem, as it has been characterized as the epidemic of the 21st century. Prevention and treatment in the initial stages of dementia are essential, and community awareness and specialization of health professionals are required, with the aim of early and valid diagnosis of the disease. Activities are recommended to the senior citizens to improve their physical and mental health. Dance has been suggested as an appropriate recreational activity for the elderly that brings functional adjustments to the various systems of the body, psychological benefits, and makes exercise to seem interesting and entertaining as it combines the performance of multiple animations with musical accompaniment. A Greek traditional dance program was performed where our sample consisted of 30 healthy elderly and 30 with Mild Cognitive Impairment – MCI. It lasted 24 weeks, two times a week for 60 min. Specific traditional dances from all over Greece were selected. The dances were of a moderate intensity at the beginning with a gradual increase in intensity, according to the age and physical abilities of the participants. The results showed a significant improvement in: attention (S4viac-Healthy: *z* = -3.085, *p* = 0.002; MCI: *z* = -3.695, *p* < 0.001, S4viti-Healthy: *z* = -2.800, *p* = 0.005; MCI: *z* = -3.538, *p* < 0.001), anxiety (Healthy: *z* = -2.042, *p* = 0.041; MCI: *z* = -2.168, *p* = 0.030), verbal fluency for MCI (Verflx: *t* = -2.396, *df* = 29, *p* = 0.023, Verfls: *t* = -3.619, *df* = 29, *p* = 0.001, Verfmo: *t* = -3.295, *df* = 29, *p* = 0.003) and in executive functions (FUCAS: *z* = –2.168, *p* = 0.030). Significant improvement also showed in physical condition (Arm curl– Healthy: *z* = –3.253, *p* = 0.001; MCI: *z* = -3.308, *p* = 0.001, Chair stand – Healthy: *t* = –3.232, *df* = 29, *p* = 0.003; MCI: *t* = -2.242, *df* = 29, *p* = 0.033, Back scratch– Healthy: *z* = -1.946, *p* = 0.052; MCI: *z* = -2.845, *p* = 0.004, 2 min step– Healthy: *z* = –2.325, *p* = 0.020; MCI: *z* = -2.625, *p* = 0.009, FootUpandGo– Healthy: *z* = -4.289, *p* < 0.001; MCI: *z* = -3.137, *p* = 0.002, Sit and Reach: *z* = -3.082, *p* = 0.002, Balance on One leg: *z* = -3.301, *p* = 0.001) and Quality of life (Healthy: *z* = -1.937, *p* = 0.053; MCI: *z* = -2.130, *p* = 0.033). This study proves that dancing not only improves the cognitive and physical condition of the elderly but also contributes to a better quality of life.

## Introduction

People living with dementia have poor access to appropriate healthcare, even in most high-income country settings, where only around 50% of people living with dementia receive a diagnosis. In low and middle-income countries, less than 10% of cases are diagnosed. As populations age due to increasing life expectancy, the number of people with dementia is increasing. We estimate that there were 46.8 million people worldwide living with dementia in 2015 and this number will reach 131.5 million in 2050 (World Alzheimer Report 2016; Prince et al., 2016). In Greece, there are more than 200,000 patients with dementia and this figure is expected to exceed to 600,000 patients by 2050, while the annual cost of dementia in Greece is now approaching six billion euros (Alzheimer Athens).

The term “dementia” is generic and refers to a complex group of changes with known or unknown etiology, which occur with widespread disruption of cognitive abilities and social functions of the individual. “Dementia can be reversible or irreversible, with rapid or slow progression, and characterized by multiple deficits of cognitive functions or almost exclusive disorder of emotion, initiative and personality” (Gorelick et al., 2011). The most common type of dementia that mainly occurs in the elderly is Alzheimer’s disease type (AD). The rapid increase in dementia ranges from about 2–3% among people aged 70–75 years and from 20–25% among people aged 85 and over (Ferri et al., 2005). The most serious and early cognitive problem in AD is memory loss. This loss is gradual and occurs within the limits of a normal level of consciousness, without any other central nervous system disorder that could explain these symptoms.

Mild Cognitive Impairment (MCI), is an emerging term that encompasses the clinical stage between normal cognitive status and dementia. It is a condition considered to be a transition between normal mental changes due to age and early clinical signs of dementia (Petersen, 2004). Its features, applications, and definitions are controversial. The MCI is now focusing on studies of natural history, biological markers and on the prevention of AD. The stage of the MCI is probably the best stage at which we could intervene with preventive strategies. Despite the conflict, progress has been made in determining the risk factors for progression from MCI to dementia. Now, treatments in order to prevent the development of AD are focusing on the MCI as a treatment group, and neurologists will increasingly be called upon to do this diagnosis. This interest is motivated by patient requests for prognosis and treatment. Therapists-neurologists have at their disposal a wealth of research information, though it is illustrated by the lack of practical suggestions for patient management (Chertkow et al., 2008). MCI is associated with an increasing risk of developing dementia. Patients with this pattern of early deficits develop dementia at a rate of 10–15% per year, while the rate for healthy controls is only 1–2% per year. However, data on the prevalence of MCI and its rate of conversion to dementia vary widely, depending on the different determinants applied.

Also, the functionality is a key area affected by aging. The functional capacity is considered to be an important part of health and wellness. The lack of mobility is the major reason that older people have problems with functionality. During aging, there are some problems in the musculoskeletal system and joints. Strength and muscle mass decrease over time. Physical activity, especially strength training, is very important action to come up against this situation (Keller and Engelhardt, 2013). There exists a small decrease in muscle strength up to 40–50 years and a 30–40% decrease in 70 to 80 years. This reduction is due to sarcopenia, which occurs more in elderly women than in men (Cruz-Jentoft et al., 2010). Genetic factors and lifestyle, such as reduction of physical activity, smoking and the use of alcoholic beverages can contribute also to sarcopenia and dementia. Muscular weakness is associated with increased risk of falls (Soriano et al., 2007; Leveille et al., 2009), resulting in possible fractures (Aniansson et al., 1984; Carpintero et al., 2014).

Physical activity stimulates the physiological functions of the body and can contribute to the stabilization of a good level of cognitive functions making the elderly more energetic. Exercise improves physical health, behavior, mental state, communication and functionality in the elderly with cognitive impairment, especially exercise that is for durability, agility, muscle strength and balance (Garber et al., 2011). The activities proposed for participation by the elderly should lead to the improvement or maintenance of physical, spiritual and mental health (Kim, 2009).

In international literature, dance has been suggested as an appropriate recreational activity for the older adults. Dance is a physical activity that causes functional changes in various systems of the human body. Previous studies have shown that elderly who dance at regular intervals have significant benefits as better balance, stability, flexibility and cognitive status than other elderly who do not dance on a regular interval (Kattenstroth et al., 2011). Dance, also offers psychological benefits and can also make the exercise more interesting and entertaining, as it combines the execution of multiple kinetic tasks with music accompaniment. For elderly, dancing is a pleasure, is exercise capacity, companionship, mental balance, wellness, coordination and muscle tone (Mullen et al., 2012). Music is still an important component of pleasure, as individuals enjoy it and express themselves through it. The rhythmic music, improves the coordination of gait and proprioceptive movement control in people with neuromuscular and skeletal disorders and leads to increased mobility and stability. Mild dance activity can prevent the risk of high blood pressure, of diabetes and the cardiovascular diseases (Gordon et al., 2004). It also helps to prevent falls and loss of bone density (Minne, 2005), improves the flexibility of the joints, especially the lower limbs, as all the muscle groups are exercised through a combination of slow and fast steps. Along with the well-being, it activates the muscles, accelerates the cardiac resistance and blood circulation, increases the burns and thus affects the metabolism, increases the maximum oxygen intake, improves the myocardial contractility, increases the frequency of breathing (Xerakia and Kalogerakou, 2000). It also requires simultaneous operation of both cerebral hemispheres, while at the same time activates kinesthetic, logical, musical and emotional processes. For this reason, dancing as a physical activity helps at a rate of 76% the risk reduction in dementia. The standard steps and specific figures does not help much. Creativity is the special component in dance that offers more results (Powers, 2010).

Greek traditional dances are an activity which offering pleasure, entertainment, education and characterized by diversity, complexity, since the combinations of lower and upper limb movements dominate and differ in intensity and in movements from other types of dance. Apart from the entertainment they offer, they are also classified as an aerobic activity that causes a burden but as part of the physiological adjustments (Galanou, 2003). Also classified as an aerobic leisure activity offering a variety of intensity and rhythm, as there is a pleasant climate during the practice (Pitsi, 2005).

In recent years, there has been growing interest in studying the quality of life. The “quality of life” is a concept with a broad scope, that is something that makes difficult its measure and its integration into the scientific study (Fallowfield, 2007). It includes epidemiological, biomedical, functional, economic and cultural approaches, as well as personal preferences, perceptions and experiences. International organizations such as the Organization of the United Nations (UN) and the World Health Organization (WHO) recognize the importance of quality of life through various declarations and conventions. Participating in properly organized exercise programs, physical education and physical activity programs contributes to positive self-esteem and high self-assessment, factors that lead to the adoption of appropriate and desirable attitudes and behaviors, greatly ensure physical well-being and mental health (Landers and Arent, 2001; Hamill et al., 2011).

The positive contribution of physical exercise to quality of life is well documented, as participation in physical activities and systematic exercise help to enhance mental well-being, increase positive mood, seek pleasurable and intense experiences, improve health and control stress, both in healthy and in clinical populations (Theodorakis, 2010). Continuously new research and work proves that exercise and participation in physical activities are associated with better performance of cognitive functions, self-esteem and self-confidence, reduction of anxiety and depression, mental well-being and an improvement in quality of life.

The aim of this study is to demonstrate the importance of Greek traditional dances in improving both the cognitive and physical health of the senior citizens. Dance is an enjoyable type of aerobic exercise that can cause various changes in the human body. We investigated if the Greek traditional dance can be an important tool for enhancing health status of senior citizens and simultaneously to improve their quality of life. Furthermore, we investigated if dance may delay the beginning of a cognitive impairment or dementia.

## Materials and Methods

### Subjects

The sample consisted of elderly people (*n* = 60) who were self-serving, had good functional and emotional state and normal or non-normal cognitive status. The subjects were divided into two groups depending on their diagnosis. More precisely, thirty participants (*n*1 = 30) were healthy seniors with median age of 65.50 years [Interquartile range (IQR) = (62.00, 68.00)] and median education of 13 years [IQR = (8.75, 16.25)] while thirty participants (*n*2 = 30) had a diagnosis of mild cognitive impairment (MCI). The MCI participants had a median age of 67.50 years [IQR = (63.00, 70.00)] and median education of 6 years [IQR = (6.00, 8.25)]. Participants did not participate in other Greek Traditional Dances programs or at any other cognitive rehabilitation programs. The intervention took place at the Greek Association of Alzheimer Disease and Relative Disorders (Alzheimer Hellas) and at the Day Care Centers of Municipality of Thessaloniki It lasted 24 weeks with a frequency of two times per week in sessions of 60 min.

Inclusion criteria were age ≥60 years, senior citizens with mild cognitive impairment, agreement of a medical doctor and time commitment to the dance protocol. Exclusion criteria were concurrent participation in another study, hypertension, heart and respiratory failure, uncorrectable vision problems, inability to participate at 80% of the hours of the program. The training program was provided at no cost and participants received no compensation. At the beginning of the program and at the end of this, a neuropsychological evaluation was performed by a psychologist to assess the cognitive, functional, and behavioral status of each participant. The fitness and functional capacity evaluated by a fitness instructor and their quality of life was assessed through appropriate questionnaires. The required descriptions were given for the purpose of this research and written consent was requested from the senior citizens to participate. Ethical and Scientific Committee of GAARD approved the protocol of this study.

### Outcome Measures

#### Psychological Evaluation

The neuropsychological assessment was performed before the intervention (initial assessment) and after the intervention of dance (final assessment). Fifteen (15) different tests were used, which examine all cognitive functions (memory, reason, judgment, abstract thinking, complex skills, attention, concentration, orientation, audiovisual perception), activities of daily living behavioral problems and quality of life. The tests that were selected are the following with a reference to what they evaluate: Mini Mental State Examination (MMSE), it is a screening instrument to separate patients with cognitive impairment from those without it (Fountoulakis et al., 2000), Clinical Dementia Rating (CDR), characterize six domains of cognitive and functional performance (Morris, 1993), Functional Cognitive Assessment Scale (FUCAS), assesses executive function in daily life activities directly in patients with dementia (Kounti et al., 2006), Functional Rating Scale for Symptoms of Dementia (FRSSD), assesses the daily functionality (Hutton, 1990), Instrumental Activities of Daily Living (IADL), assesses the independent living skills (Theotoka et al., 2007), Test of Every Day Attention (TEA), assesses the attention (Robertson et al., 1996), Trail-making Test (TMT), assesses the executive function (Vlahou and Kosmidis, 2002), Rey–Osterreith Complex Figure Test (ROCF), assesses the memory (Rey and Osterrieth, 1993), Rey Auditory Verbal Learning Test (RAVLT) and Rivermead Behavioral Memory Test (RBMT), assesses the memory (Efkildes et al., 2002), Verbal Fluency Test (VFT), assesses the cognitive function (Kosmidis et al., 2004), Neuropsychiatric Inventory (NPI), assesses the range of neuropsychiatric symptoms (Politis et al., 2004), Geriatric Depression Scale (GDS), assesses the depression (Fountoulakis et al., 1999), Quality of Life in Alzheimer’s Disease (QOL-AD), assesses the quality of life (Logsdon et al., 1999), Beck Anxiety Inventory (BAI), record the anxiety (Beck and Steer, 1988). These tests have been selected on the basis of the validity and reliability and the existence of norms for the Greek population (Kosmidis, 2008; Tsolaki and Kounti, 2010).

#### Physical Evaluation

In order to assess their physical condition and functional capacity, the Body Mass Index (BMI) was first calculated and then the Senior Fitness Fullerton Test was used, which consists of six tests and evaluates the flexibility of low back and hamstrings, the functional capacity of individuals through the strength of the lower limbs and the dynamic balance, the speed, agility and balance during movement (Jones and Rikli, 2002). In addition, their static equilibrium was evaluated through the Flamingo test (Barabas et al., 1996), the length of time spent on one leg was calculated, the strength of the strong hand was recorded with the use of the dynamometer (Saehan Corp., Masan, South Korea) and the jumping ability (vertical jump) was evaluated using OptoJump system (Microgate, Bolzano, Italy). Specifically, the tests used are: Chair stand, 8 FootUpandGo, Back Scratch, Arm Curl, Chair Sit and Reach, 2 Min Step, Balance One leg, Hand Grip Strength, and Jumping ability.

#### Quality of Life Evaluation

To evaluate the quality of life, the questionnaire developed by the WHO was used, the WHOQOL, which aims to promote an intercultural Quality of Life assessment system and the use of this questionnaire in the wider health sector. It includes 26 questions and is divided into four thematic sections (Skevington et al., 2004) where the relevant questions address: (a) physical health; (b) mental health; (c) social relations; and (d) the environment. It also includes two questions, which offer an overall assessment of Quality of Life and Health Status (Ginnieri-Kokkosi et al., 2003). The results with the highest values are an indication of a better quality of life. In general, the multifaceted Quality of Life is examined, as well as a general state of health.

#### Selection of Greek Traditional Dances

The traditional dances selected from all over Greece. The design of the program held by dividing the dances into three categories depending on the complexity and number of steps, the rate of intensity (slow speed) and the position-movement of the hands. Dances were also classified into three categories: mild, moderate and high intensity. Most of them were in moderate intensity, with progressive and increasing intensity, indicative of the age and physical abilities of the participants.

### Statistical Analysis

#### Demographics

We planned comparisons between the independent variables age and education level between groups, respectively. Initially, demographic data were tested for normality assumption between groups (Healthy, MCI) using visual inspection of histograms, normal Q-Q plots and boxplots, in terms of Skewness and Kurtosis as well as using the normality tests (Shapiro and Wilk, 1965; Razali and Wah, 2011). If the independent variable was approximately normally distributed in both groups, differences between groups were explored using parametric methods (independent samples *t*-tests). However, if the normality assumption was not met, non-parametric analysis (Mann–Whitney *U*-Test) was followed. Additionally, the possible association between and the gender (male, female) and the group (Healthy, MCI) was investigated by means of Chi-squared test.

The participants’ demographic information was described in tables in terms of mean (standard deviation) or median, interquartile range, respectively, depending on the normality assumption. More precisely, when normality assumption was met, the mean (standard deviation) was used whereas in non-normally distributed variables, median and interquartile range was depicted.

Statistical analysis was performed using the IBM SPSS Statistics (Version 20) and the level of significance was set at *p* < 0.05.

#### Data

Tasks examining the neuropsychological and somatometric state as well as the quality of life of the participants were performed both before and after the intervention in both groups (Healthy and MCI participants). As assumptions for a Mixed Model Analysis of Variance (or Split-plot ANOVA) were not fulfilled, an alternative analysis was performed. Differences in scores collected from the neuropsychological and somatometric assessment at the two-time points (after training – before training scores) were computed and then tested for normality. The within-group changes, after grouping our data by diagnosis, were explored using either paired *t*-test or Wilcoxon signed-rank test depending on normality assumption of score differences at the two-time points. Additionally, the between-group differences were explored comparing score differences between the two groups using either independent samples *t*-test or Mann–Whitney *U*-test based on normality assumption of score differences. The methodology used has been published elsewhere (Cramer, 1998; Cramer and Howitt, 2004; Doane and Seward, 2011; Arvanitidou-Vagiona and Xaidits, 2013; Athanasiou et al., 2017; Pandria et al., 2018).

## Results

### Demographics

Both variables age and education were not approximately normally distributed for both groups (Healthy, MCI).

Planned comparisons between groups revealed that the age did not significantly vary between healthy and MCI participants (*U* = 333.500, *p* = 0.084) whereas MCI individuals seem to have significantly fewer educational years compared to healthy participants (*U* = 183.500, *p* < 0.001) (Table 1). The proportion of male/female (6/24) participants were equal for both groups and as such no significant association was found between gender and group (χ^2^= 0.000, *df* = 1, *p* = 1.000).

**Table 1:** Demographic data as age and education of healthy and MCI participants.

Groups	Median, interquartile range (IQR)	
	Age	Education
Healthy (30 elderly)	65.50, 6.00	13.00, 8.00
Mild cognitive impairments-MCI (30 elderly)	67.50, 7.00	6.00, 2.00

### Neuropsychological Data

The performance of healthy and MCI participants significantly changed at the subtests of TEA test, S4viac (Healthy: *z* = -3.085, *p* = 0.002; MCI: *z* = -3.695, *p* < 0.001) and S4viti (Healthy: *z* = -2.800, *p* = 0.005; MCI: *z* = -3.538, *p* < 0.001). More precisely, both healthy and MCI participants showed significant improvement in S4viac test [Healthy – Before training: 9.00 (5.00, 10.00); After training: 10.00 (9.75, 10.00); MCI – Before training: 6.50 (4.00, 10.00); After training: 10.00 (8.00, 10.00)]. However, a significant decrease was observed in S4viti test for both groups [Healthy – Before training: 6.32 (5.20, 9.47); After training: 5.36 (4.36, 6.50); MCI – Before training: 8.58 (6.03, 11.70); After training: 6.39 (5.36, 7.72)].

Additionally, significant decreases were found in RBMT1 and RBMT2 tasks for both groups [RBMT1: Healthy – Before training: 14.00 (11.75, 15.00); After training: 12.00 (9.13, 15.00); *z* = -3.176, *p* = 0.001; MCI – Before training: 11.00 (9.00, 13.00); After training: 8.00 (5.88, 10.00); *z* = -3.811, *p* < 0.001; RBMT2: Healthy – Before training: 12.25 (10.00, 15.00); After training: 12.00 (7.88, 15.00); *z* = -1.986, *p* = 0.047; MCI – Before training: 10.00 (6.00, 13.00); After training: 6.25 (4.00, 10.00); *z* = -3.580, *p* < 0.001]. Moreover, anxiety levels have found to be considerably altered, as measured by the BAI test (Healthy: *z* = -2.042, *p* = 0.041; MCI: *z* = -2.168, *p* = 0.030). More precisely, MCI individuals showed improvement in anxiety levels based on the scores in BAI test when comparing their scores in two-time conditions [Before training: 7.50 (3.00, 12.25); After training: 4.50 (2.00, 10.25)] whereas anxiety levels in healthy participants were increased [Before training: 2.50 (1.00, 6.50); After training: 4.00 (1.00, 8.50)]. In contrary to this, when comparing the MCI participants’ scores in two-time conditions at PSS test they showed significant increase in their scores [Before training: 6.63 (6.75); After training: 9.73 (5.92); *t* = -2.168, *df* = 29, *p* = 0.024].

**FIGURE 1:**
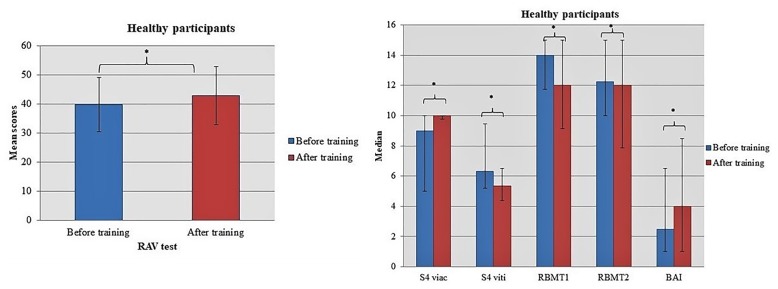
Significant design. differences in the performance of Healthy participants when comparing tests’ scores in two-time conditions. Asterisk indicates the *p*-values that reached statistical significance (*p* < 0.05).

Healthy individuals seem to improve their immediate memory and delayed recall as they scored higher at RAV test [Before training: 39.77 (9.36); After training: 42.87 (10.08); *t* = 2.095, *df* = 29, *p* = 0.045] in the post-intervention screening (Figure 1). On the other hand, MCI participants benefited the most from dancing to verbal fluency scoring higher at tasks Verflx [Before training: 7.97 (3.10); After training: 9.33 (3.19); *t* = -2.396, *df* = 29, *p* = 0.023], Verfls [Before training: 8.03 (3.08); After training: 10.00 (3.33); *t* = -3.619, *df* = 29, *p* = 0.001] and Verfmo [Before training: 8.21 (3.02); After training: 9.54 (2.99); *t* = -3.295, *df* = 29, *p* = 0.003].

**FIGURE 2:**
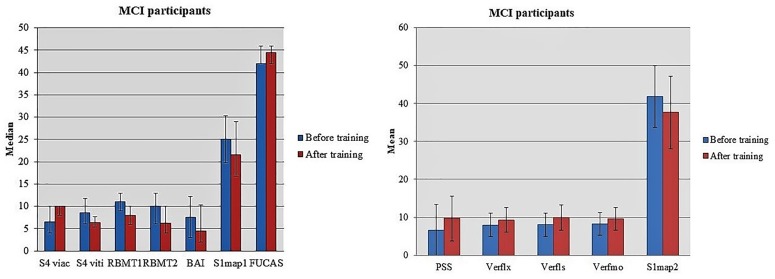
Statistically significant alterations in the performance of MCI participants when comparing tests’ scores in two-time conditions.

Considerable deviations in the performance of MCI individuals at S1map1 [Before training: 25.00 (19.75, 30.25); After training: 21.50 (16.75, 29.00); *z* = -2.153, *p* = 0.031] and S1map2 [Before training: 41.87 (8.11); After training: 37.67 (9.57); *t* = 2.508, *df* = 29, *p* = 0.018] tasks have observed. Marginally significant difference was found in scores of MCI group at NPI task (*z* = -1.912, *p* = 0.056) whereas a statistically significant decrease was reported in their functionality based on FUCAS test [Before training: 42.00 (42.00, 46.00); After training: 44.50 (42.00, 46.00); *z* = -2.168, *p* = 0.030] (Figure 2).

**FIGURE 3:**
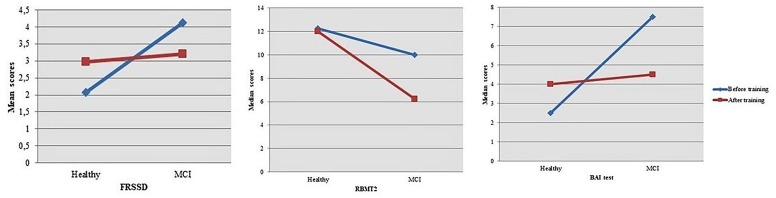
Significant interactions time × group were found when comparing post-pre-differences in scores at FRSSD, RBMT2, and BAI tests between the two groups.

Furthermore, we performed comparisons of score differences in two-time conditions (post-pre-scores) between groups resulting in significant interactions time × group at FRSSD [Healthy: 0.9 (2.56); MCI: -0.93 (2.64); *t* = 2.729, *df* = 58, *p* = 0.008], RBMT2 [Healthy: -1.00 (–2.63, 0.25); MCI: -2.00 (–5.00, 0.00); *U* = 312.500, *p* = 0.041] and BAI [Healthy: 0.00 (–0.25, 4.00); MCI: -2.00 (–5.00, 1.00);*U* = 262.000, *p* = 0.005] tests (Figure 3). Based on the aforementioned results, MCI participants showed greater improvement in FRSSD and BAI tests.

### Somatometric Data

**FIGURE 4:**
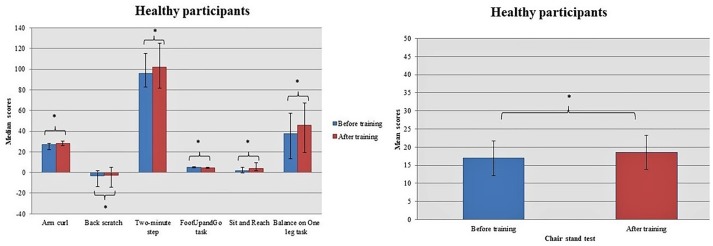
Healthy participants improved their performance in most of the somatometric tests. Asterisk indicates the *p*-values that reached statistical significance (*p* < 0.05).

**FIGURE 5:**
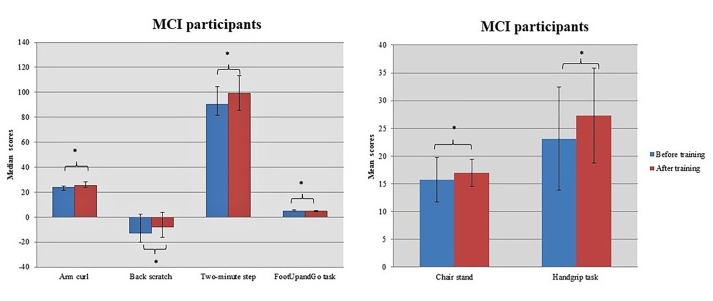
MCI participants benefited most from the intervention in the tasks Chair stand, Handgrip, FootUpandGo, Back scratch, Arm curl, and the Two-Min step. Asterisk indicates the *p*-values that reached statistical significance (*p* < 0.05).

Both groups showed considerable improvement after dancing in their strength of both upper [Arm curl task: Healthy – Before training: 27.00 (22.00, 28.25); After training: 28.00 (25.75, 30.25); *z* = -3.253, *p* = 0.001; MCI – Before training: 24.00 (21.50, 25.00); After training: 25.50 (24.00, 28.00); *z* = -3.308, *p* = 0.001] and lower limbs [Chair stand task: Healthy – Before training: 16.97 (4.80); After training: 18.57 (4.76); *t* = -3.232, *df* = 29, *p* = 0.003; MCI – Before training: 15.73 (4.02); After training: 16.97 (2.46); *t* = -2.242, *df* = 29, *p* = 0.033] as well as in the flexibility of the shoulder belt [Back scratch task: Healthy – Before training: -3.00 (–13.75, 2.00); After training: -2.50 (-14.25, 5.00); *z* = -1.946, *p* = 0.052; MCI – Before training: -13.00 (-20.00, 2.25); After training: -8.00 (–16.25, 4.00); *z* = -2.845, *p* = 0.004]. Additionally, the intervention seems to promote gains in aerobic capacity [2-min step: Healthy – Before training: 96.00 (82.75, 115.25); After training: 102.00 (81.50, 125.00); *z* = -2.325, *p* = 0.020; MCI – Before training: 90.50 (81.75, 104.50); After training: 99.00 (85.50, 113.50); *z* = -2.625, *p* = 0.009] and movement coordination [FootUpandGo task: Healthy – Before training: 4.89 (4.36, 5.78); After training: 4.41 (4.18, 5.16); *z* = -4.289, *p* < 0.001; MCI – Before training: 4.99 (4.53, 5.70); After training: 4.73 (4.13, 5.14); *z* = -3.137, *p* = 0.002] for both health and MCI individuals (Figures 4, 5). Moreover, healthy participants improved their suppleness of back and the lower femoral back along with their balance achieving higher scores at Sit and Reach task [Before training: 2.00 (–0.25, 5.00); After training: 4.00 (1.50, 9.25); *z* = -3.082, *p* = 0.002] and Balance on One leg task [Before training: 37.28 (13.46, 57.30); After training: 45.76 (19.54, 67.06); *z* = -3.301, *p* = 0.001], respectively. On the other hand, MCI individuals marginally altered their performance at the Handgrip task [Before training: 23.13 (9.27); After training: 24.27 (8.54); *t* = 2.014, *df* = 29, *p* = 0.053].

**FIGURE 6:**
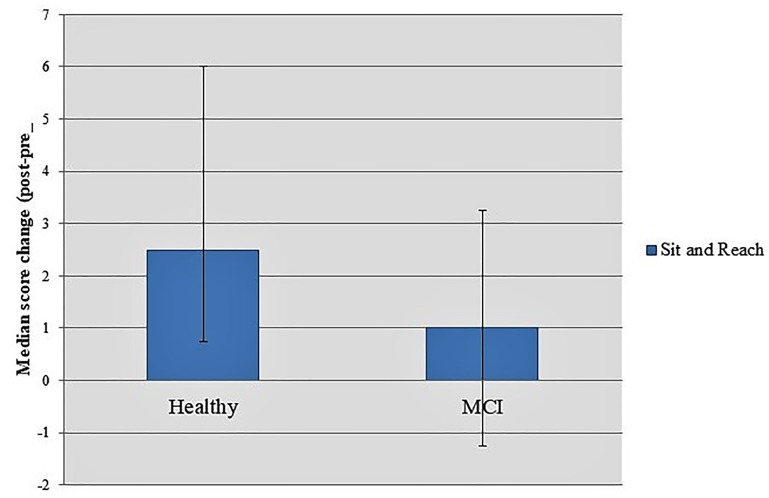
Score differences in two-time conditions between groups showed a marginally significant interaction time × group in Sit and Reach task.

Planned comparisons of score differences in two-time conditions between groups revealed a marginally significant interaction time × group in Sit and Reach task [Healthy: 2.50 (0.75, 6.00); MCI: 1.00 (–1.25, 3.25); *U* = 322.000, *p* = 0.057] (Figure 6).

### Quality of Life Parameters

**FIGURE 7:**
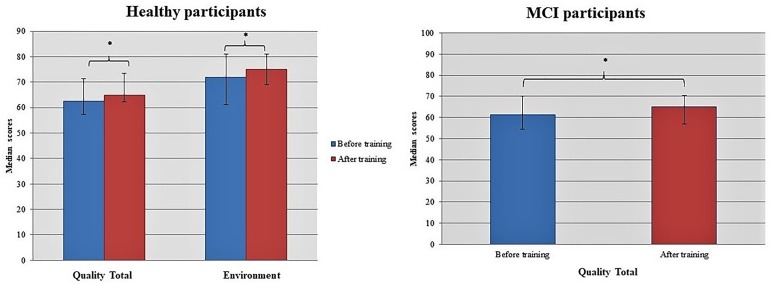
Dancing seems to promote generally significant changes in the quality of life of both healthy and MCI participants. Asterisk indicates the *p*-values that reached statistical significance (*p* < 0.05).

Dance seems to promote generally significant gains in the quality of life and health status for both healthy [Before training: 62.63 (57.25, 71.25); After training: 64.88 (62.13, 73.56); *z* = -1.937, *p* = 0.053] and MCI [Before training: 61.25 (54.44, 70.13); After training: 65.00 (57.06, 70.50); *z* = -2.130, *p* = 0.033] individuals (Figure 7). Moreover, healthy participants due to the intervention improved their interaction with the environment [Before training: 72.00 (61.25, 81.00); After training: 75.00 (69.00, 81.00); *z* = -2.062, *p* = 0.039].

## Discussion

In this research, it is observed that the dance intervention has presented significant benefits to mental and physical health in healthy elderly and in elderly with MCI, also in their quality of life. Their performance significantly changed in the assay examining the daily attention (selective attention, sustained attention, stirring of attention and execution of dual work in visual and auditory attention). Specifically, in two sub-tests, significant statistical results were observed. It seems that the effect of dance was positive in this particular test. The test requires in terms of the participant not only speed but also good visual competence. Consequently, the effect of dance seems to be particularly beneficial to participants, increasing their alertness and improving their visual perception. In addition, intervention has altered the performance of visual and audio information, and the level of anxiety was found to have changed significantly. In particular, Mild Cognitive Impairment (MCI) individuals showed an improvement in their anxiety levels based on their two-time scores, while healthy participants increased stress levels. Healthy individuals appear to have improved their immediate and delay memory as they were scored higher in the post-intervention test. This increase indicates, that after the dance intervention participants were able to improve their memory levels. The fact that dance intervention has had positive effects on memory improvement is considered to be particularly important because the most common form of dementia is AD, which basically decreases the brain hippocampus resulting in memory difficulties. The speech fluency seems to be dwindling as dementia progresses, so patients cannot even say a word in advanced stages of the disease. In the final count, the number of words recalled in each sub-test of the verbal option was significantly larger than the original one. In our research, it was observed that the dance had a positive effect on the healthy group as it improved their performance, but the effects were more admirable in the MCI participants. This shows that dance has produced positive results, which helped them to function and think faster. All of these findings, are based on the effectiveness of dance in elderly people and in particular in their physical and mental health, as shown by other studies (Kim et al., 2011; Kattenstroth et al., 2013).

The criterion for the separation of MCI from dementia is the ability to resolve daily activities, something that patients with MCI can achieve with a potentially relatively slower pace than normal elderly, but they can achieve it with positive results, while patients with dementia, even at baseline, seems to be unable to do so. After the dance intervention, the price seems to have increased. This could be explained in two ways: (a) Probably the increase in units in the test is a normal increase, as we know that dementia is an evolving disease and therefore over time it is expected that the patient will have more difficulty and (b) dance can maintain the functionality of the patients which would statistically worsen if they did not take the dance intervention. As shown in Hwang and Braun (2015), survey, adding physical activity to one’s life is an effective method of preventing, controlling, and alleviating some health conditions. Studies have demonstrated that physical activity has positive effects on depression, anxiety, dementia, heart failure, stroke, cognition and sleep. The harmful effects resulting from physical inactivity and the positive effects of physical activity suggest that further efforts are needed to encourage physical activity, with an emphasis on populations at high risk for inactivity. Maintaining the functional capacity of the elderly at satisfactory levels, is something that leads them to an independent and quality lifestyle, while reducing the risk of various diseases. In our research, the two groups showed a significant improvement after the intervention of the dance in their strength both in the upper and low part of the body, as well as in the flexibility. Moreover, the dance seems to promote significant benefits to their aerobic capacity and coordination of movements, both in healthy and in MCI individuals.

In addition, healthy participants improved their flexibility in the lower back and in femoral back as well as their equilibrium by achieving higher scores. Both teams have improved their functional capacity and body balance, especially the skills that related to day-to-day activities such as luggage or shopping and they have gained more confidence and independence as physical strength and energy allow them to engage in more activities with less fatigue.

Controlling the balance and maintaining the strength of the lower limbs, are considered important in order to reduce the episodes of drastic falls (Buchner et al., 1997). Many investigations have reported the importance of the ankle joint in the entire human body’s mechanics, particularly in sensitive groups such as the elderly, in whom the muscles around the joint seem to be more affected by old age. As the strength of the dorsal flexors of the ankle seems to be affected by aging, especially in the elderly with a history of falls, it is likely that the strengthen of this particular muscle group improves the control of the elderly, resulting in falls reduction. Balance is an important functional capacity that influences significantly the ability of human to perform daily activities for their survival, such as maintaining a stable posture, the straight movement from one position to another and maintain the upright posture (Islam et al., 2004).

Quality of life refers not only to one or some particular external features, but rather expresses an existential state of the individual. Its assessment methodology should focus on identifying those factors that have a particular focus on subjective judgment and quality of life assessment. The results of our research showed statistically significant effects in the overall quality of life and the general health status of participants in the Greek traditional dances program. Dance generally seems to promote significant gains in quality of life and health status for both the healthy and for the MCI subjects. In addition, healthy participants due to dance intervention improved their interaction with the environment. It appeared that they began to acquire a sense of security with regard to external dangers, they have also started to engage in various recreational activities in their spare time and to use the means of transport more easily. Cruz-Ferreira et al. (2015), presented the positive effects of dance in different dimensions of functioning and the potential to contribute to healthy aging. This could be related to the integrated mobilization of physical, cognitive, and social skills promoted by creative dance. Also, Sivvas et al. (2015) at his study, shown that dancing helps in many ways to preserve and improve human health, as far as physical health is concerned – as it maintained the physical state in good level, but also concerning mental health – by minimizing stress and depression. Finally, social health also proved to be positively affected as the factors that prevent an individual from socialization were reduced.

Generally, our findings are in line with Teri et al. (2008) study that for long-term participation of dementia patients in exercise programs, it is necessary to be able to perform them, be fun and enjoyable. Thus, the pleasant environment, the effective communication, the feeling of security as well as the entertaining character of the exercise programs, strongly consent to the regular participation of patients in them.

In Greece than in many other countries, there is a “living tradition,” i.e., in the villages living tradition continues, evolves and sometimes consciously experienced. This is an element of the inextricable relationship of the Greeks with their traditions that are lost in the depths of the ages. Traditional dances are deeply rooted in the culture of Greece, expressing local history, traditions and customs, express culture. By learning the dances of an area, someone could learn at the same time its peculiarities, history, geographic stories and myths. It is a necessity for the Greek people to remain in tradition, with the result that local dances have a strong expression of emotions but also creativity. We could investigate dances from different countries in a next study in order to evaluate the results that will arise.

## Conclusion

The participation of the elderly in activities helps them to maintain their physical status, but also gives them the opportunity to interact with other people of all ages. This interaction, removes the feeling of loneliness, which stimulates their psychological state. Also, improves their self-esteem as they realize that they can participate in new skills. Dance as exercise, increases body resistance, helps them to maintain proper posture, stimulates the muscular system and improves physical fitness. Combined with music, helps to express emotions, combat stress and improves mental health. With the repetition of the steps, the movement of the hands and their combined function helps to improve the cognitive functions.

Furthermore, dance seems to be particularly important to protect against dementia and to slow the progression of the disease. Socialization contributes to the maintenance of positive psychology, which can be a protective shield for dementia, and to protect patients with MCI from potential depression, which adds to the situation of a patient with mental problems. It is true that nowadays, the non-pharmaceutical interventions for the treatment of dementia play a particularly important role on the world stage, and our research confirms the studies that have been established so far which mention the importance of dance.

## Future Directions

The results of our research suggest that the intervention of Greek traditional dances in elderly is effective for their physical and mental health. Future research should focus on an additional diagnostic test such as neuropsychological recordings through an electroencephalography that records the brain’s functions and in particular its electrical activity. After the end of interventional assessment will identify any changes that occur in brain activity. Generally, the electroencephalography is a very useful method, because it can non-invasively and painlessly give a complete view of brain function or even brain disorders.

## Author Contributions

VZ designed and implemented the dance program, collected and analyzed the data, prepared the initial draft of the manuscript, guided the analysis, and revised the manuscript. OL implemented the dance program, collected the data, and revised the manuscript. MT guided the study. SD co-guided the study.

## Conflict of Interest Statement

The authors declare that the research was conducted in the absence of any commercial or financial relationships that could be construed as a potential conflict of interest.
